# Supplementary material for: Post-transcriptional 3´-UTR cleavage of mRNA transcripts generates thousands of stable uncapped autonomous RNA fragments
Source: Nat Commun. 2017 Dec 11;8:2029. doi: 10.1038/s41467-017-02099-7 (PMC5725528; doi:10.1038/s41467-017-02099-7)
Supplement: Supplementary file 1 — Supplementary Information [file 41467_2017_2099_MOESM1_ESM.docx]

**Supplementary Figures:**

| 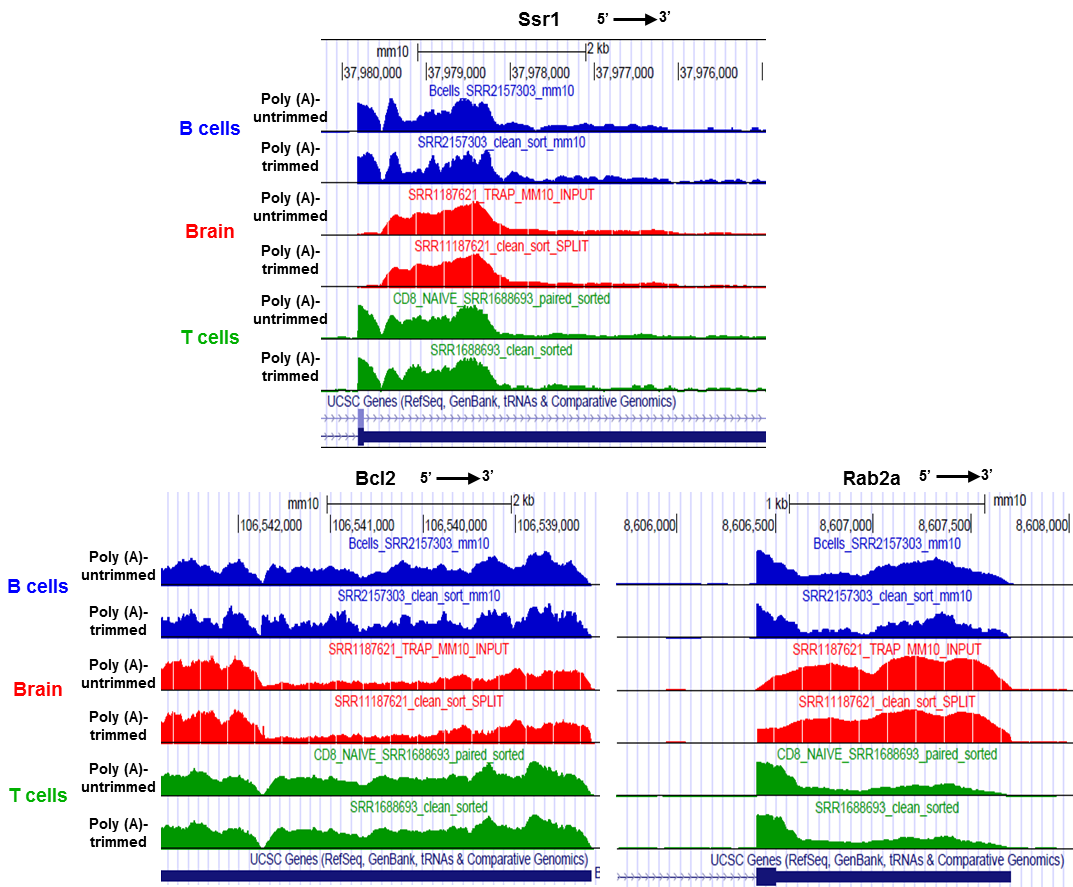 |
| --- |
| **Supplementary Fig. 1** RNA-seq read coverage for Ssr1, Bcl2, and Rab2a in mouse T cells, B cells, and brain tissue. To exclude the possibility that observed gaps in coverage are due to poor mapping following RNA polyadenylation, we compared the coverage without (top) or with (bottom) trimming of poly(A) runs at read ends. |

| **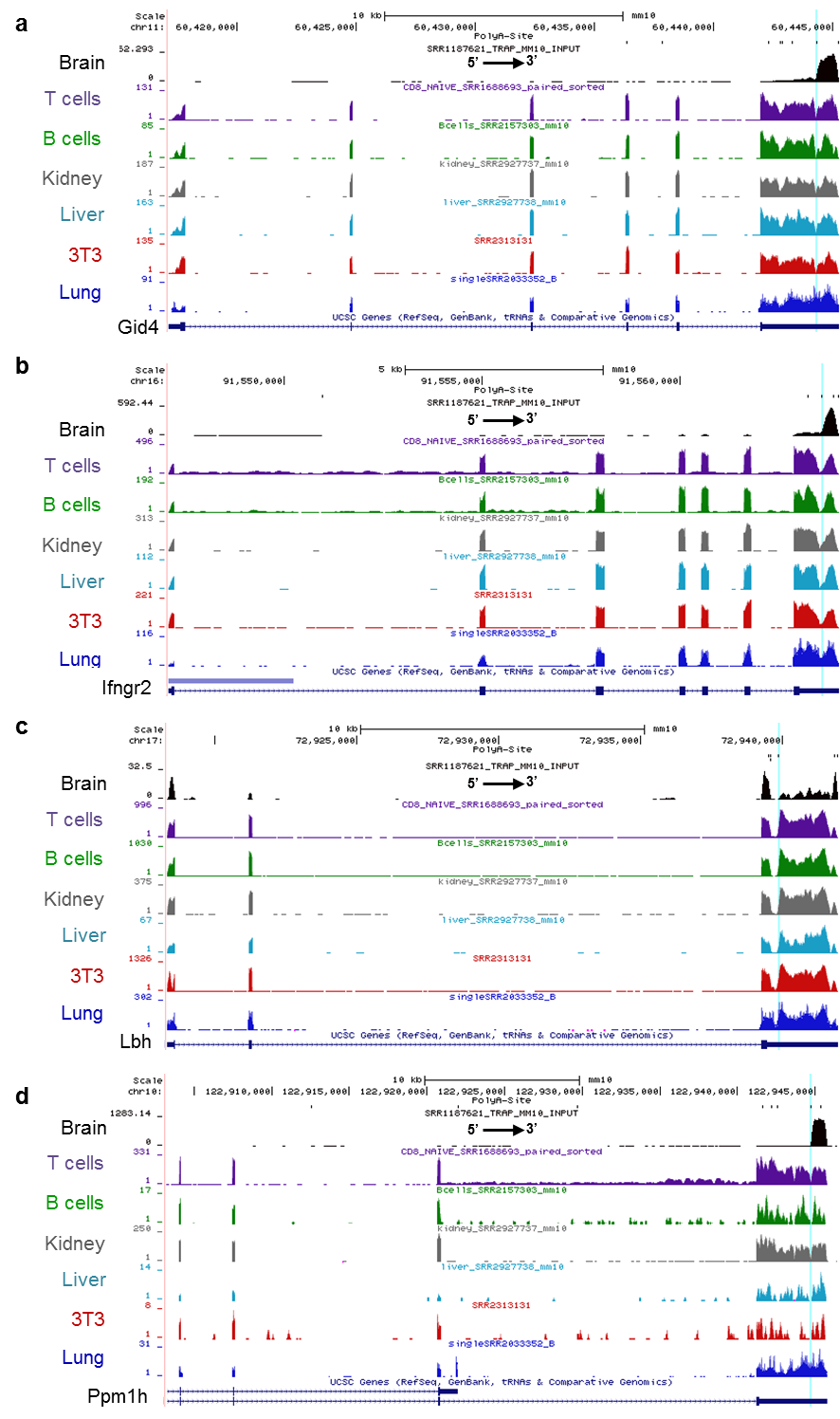** |
| --- |

| **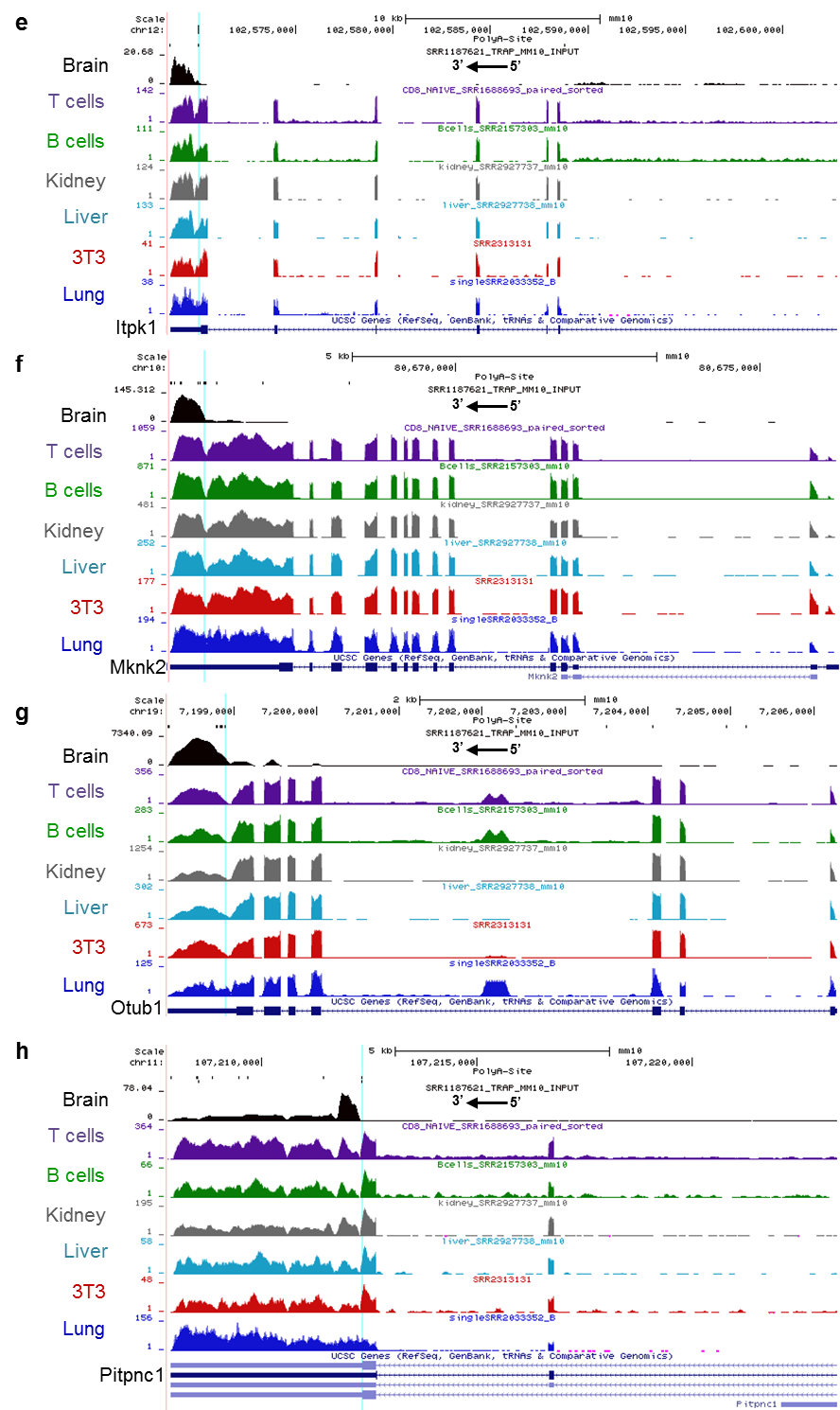** |
| --- |

| **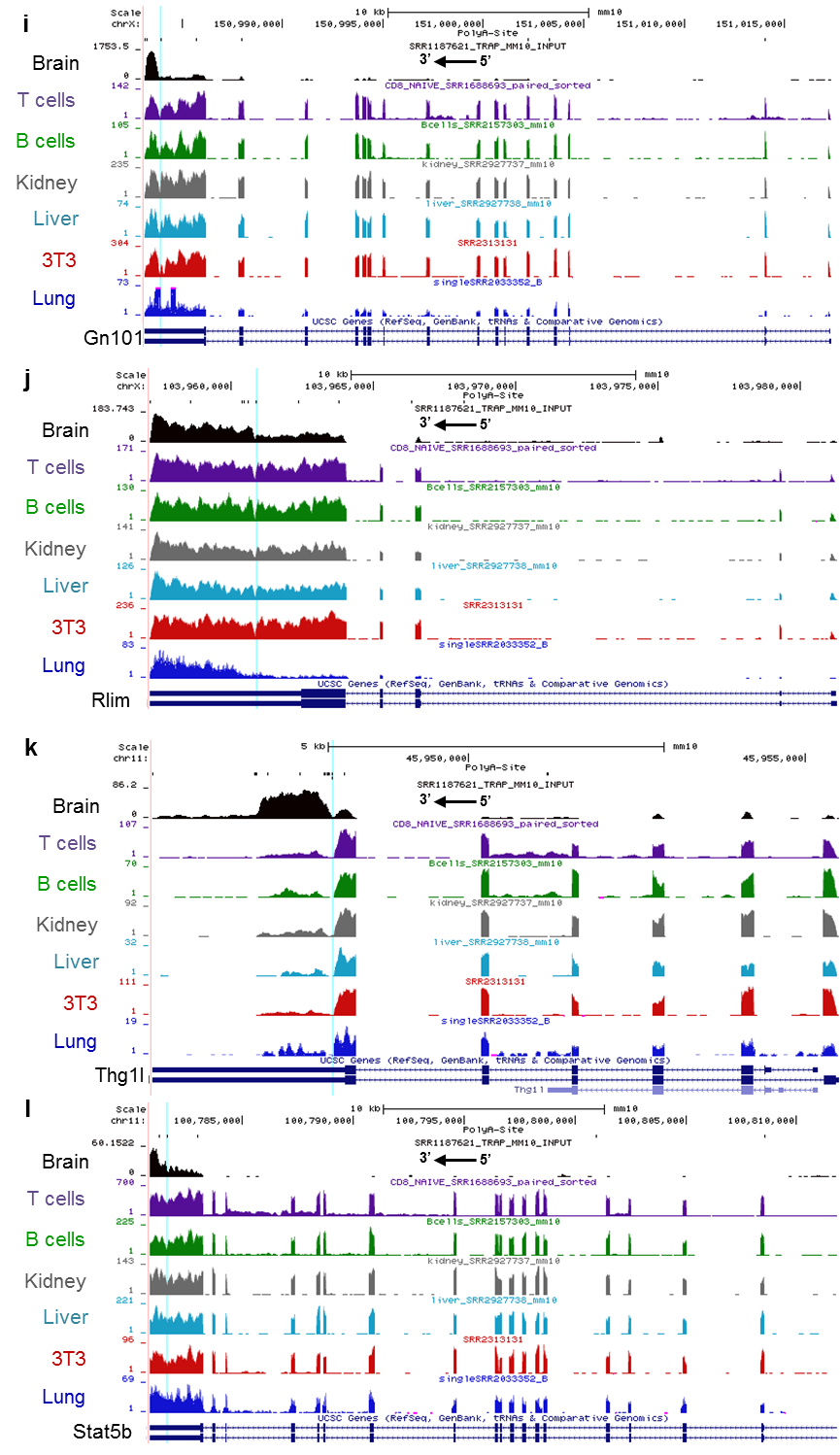** |
| --- |

| **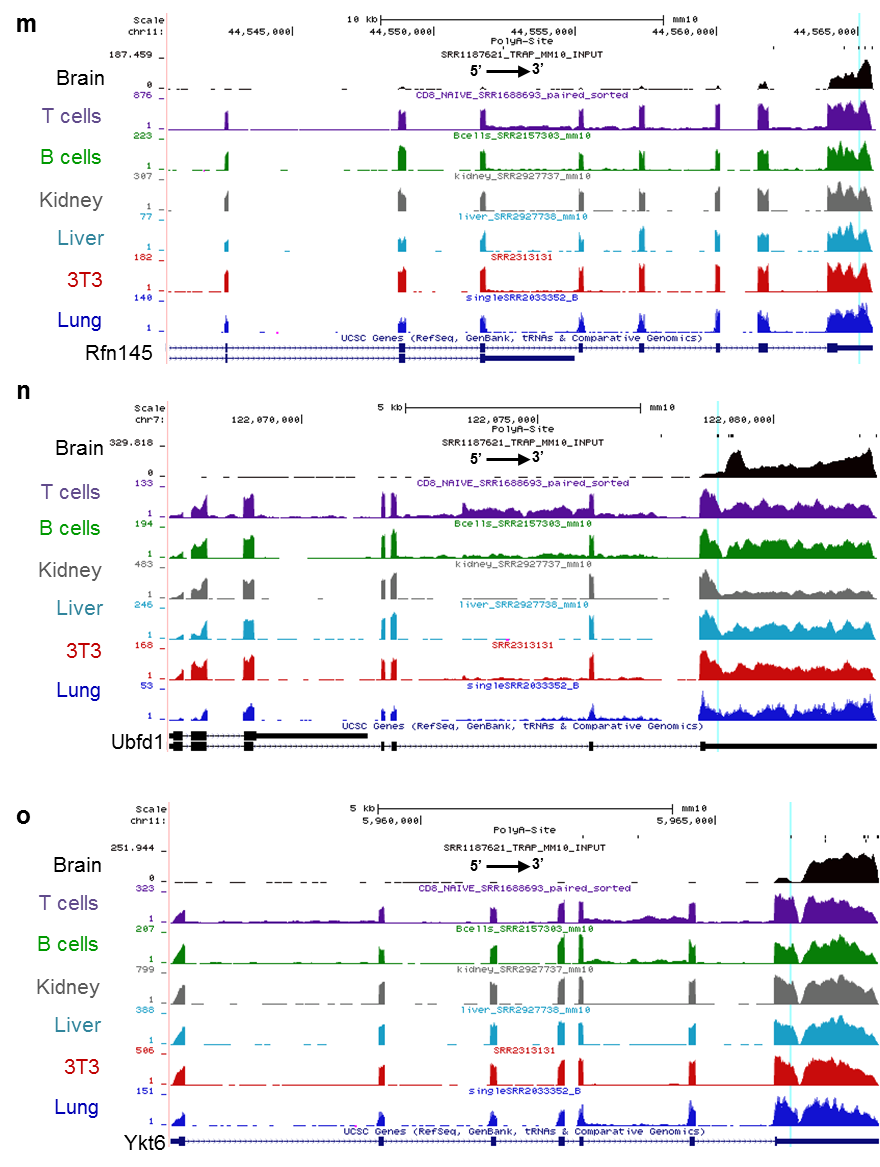** |
| --- |
| **Supplementary Fig. 2**  RNA-seq read coverage for Itpk1, Mknk2, Otub1, Pitpnc1, Gid4, Infgr2, Lbh, Ppm1h, Gn101, Rlim, Thg1l, Stat5b, Rfn145, Ubfd1 and Ykt6 in mouse brain tissue ([SRX484869](https://www.ncbi.nlm.nih.gov/sra/SRX484869%5baccn%5d)), T cells (SRX793419), B cells (SRX1144018), Kidney (SRX1421800), liver (SRX142180), 3T3-NIH (SRX1210977) and lung (SRX1033584), demonstrating a gap in the 3’UTR (marked by light blue line), as well as uneven levels of RNA upstream and downstream of the gap site. |

| 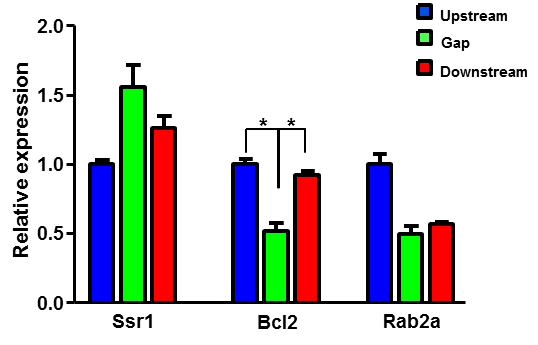 |
| --- |
| **Supplementary Fig. 3** qRT-PCR analysis of upstream, gap and downstream regions from total RNA extraction from naïve T cells of Ssr1, Bcl2 and Rab2a genes. *P<0.05, (two-tailed Student’s t-test). Results are representative of three independent experiments. Error bars, s.d. |

| 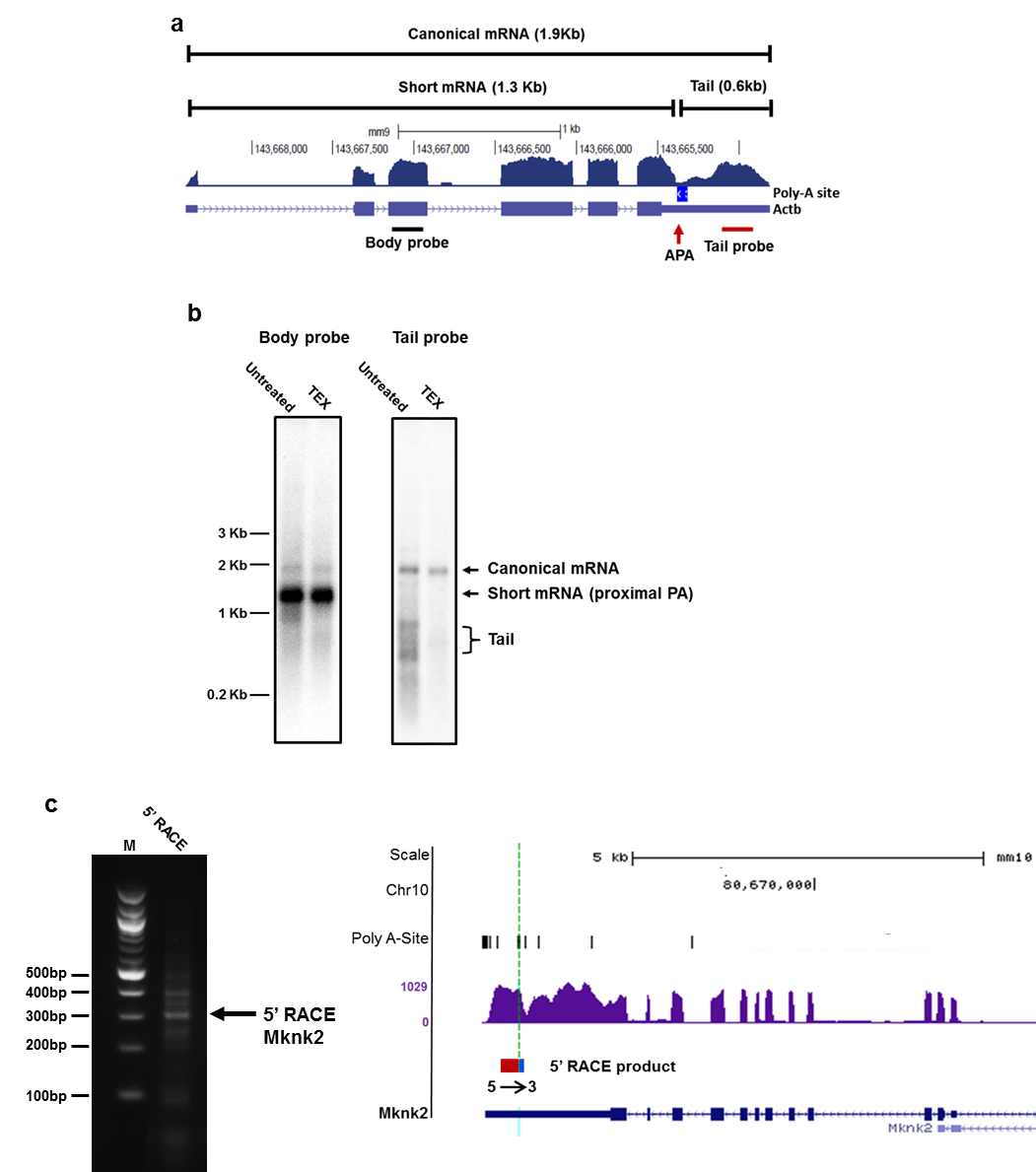 |
| --- |
| **Supplementary Fig. 4** Northern blot analysis of Actb RNA (**a**-**b**) and 5’RACE analysis of Mknk2 RNA (**c**). **a** Actb presented APA site corresponds to a cleavage in RNA-seq data from mouse heart (SRX1421799). **b** Northern blot of Actb. 0.5µg of PolyA+ RNA separated on 1.6% agarose from mouse heart. Riboprobes for CDS (body) and 3’UTR (tail) were used in control RNA samples and after TEX treatment. **c.** 5’RACE: Sequencing PCR product of 5’ Rapid amplification of cDNA ends of Mknk2 identified the 5’ end of 3’UTR tail fragment is in correspondence with APA site. Red box: mapped sequence to the corresponding gene. Blue box: 5' RACE annealed oligonucleotide. See Supplementary Data 2 for the sequences of the 5’RACE product. |

| 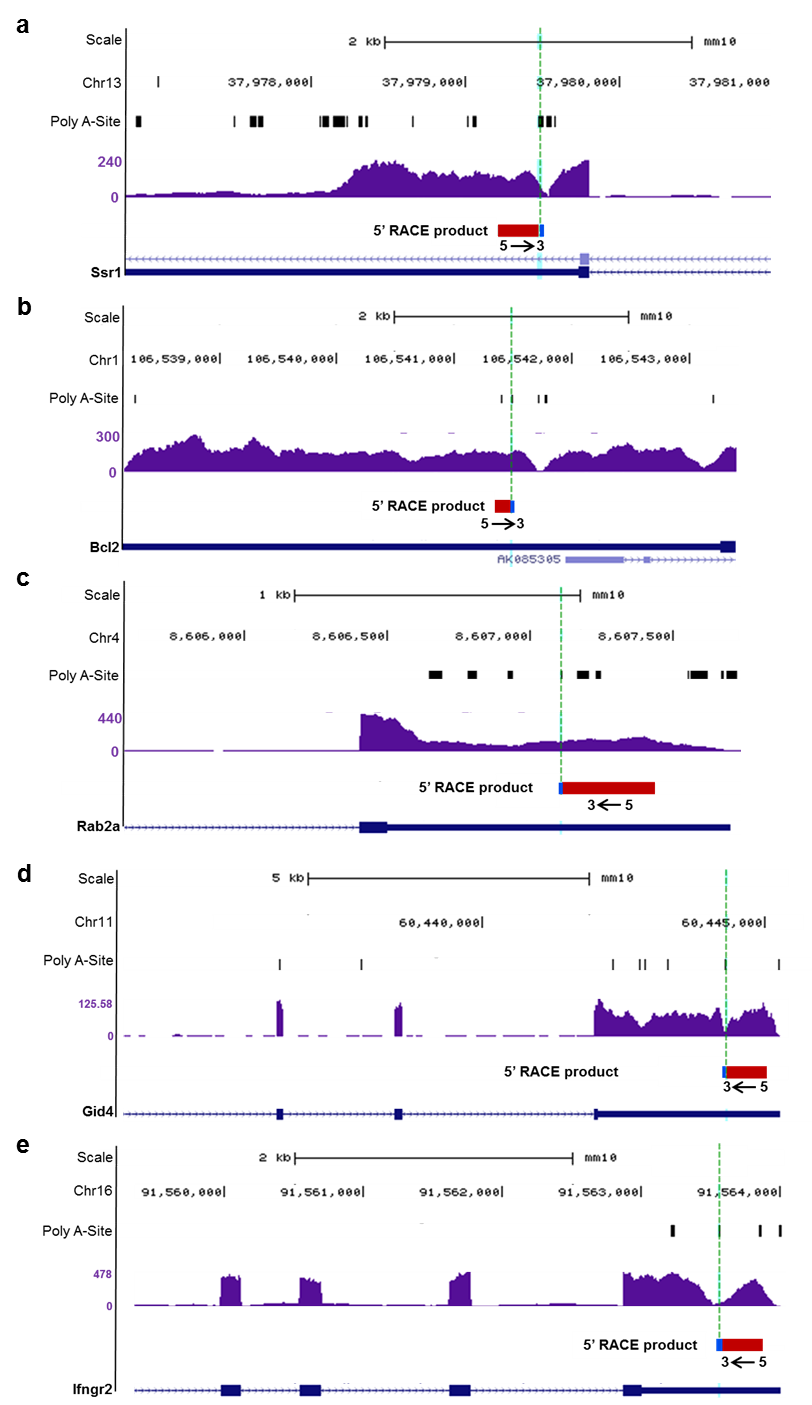 |
| --- |
| **Supplementary Fig. 5 a-f** 5’RACE: PCR products of 5’ Rapid amplification of cDNA ends of Ssr1, Bcl2, Rab2a, Gid4 and Ifngr2 were sequenced. 5’ end of 3’UTR tail fragment is mapped adjacently to APA site. Red box: mapped sequence to the corresponding gene. Blue box: 5’ RACE annealed oligonucleotide. See Supplementary Data 2 for the sequences of the 5’RACE products. |

| 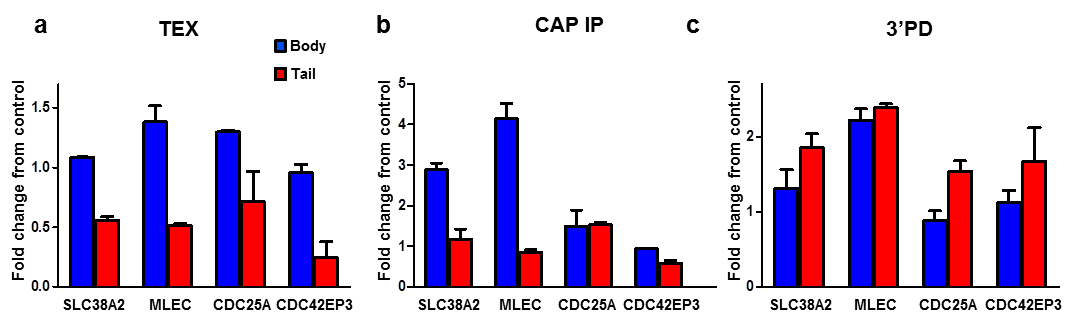 |
| --- |
| **Supplementary Fig. 6 a** qRT-PCR measurements of body and tail after polyA selection and treatment with TEX. **b** qRT-PCR measurements of body and tail after polyA selection and immunoprecipitation with anti-CAP antibody. **c** Vaccinia Capping System was used to cap an uncapped RNA with an addition of biotinylated GTP. qRT-PCR measurements of body and tail after polyA selection and pulldown with Streptavidin beads for a biotinylated capped RNA. Expression levels were normalized to Ubc and the body fragment was used as a reference to expression level. *P < 0.05, **P < 0.01 and ***P < 0.001 (two-tailed Student’s t-test). Results are representative of three independent experiments. Error bars, s.d. |

| 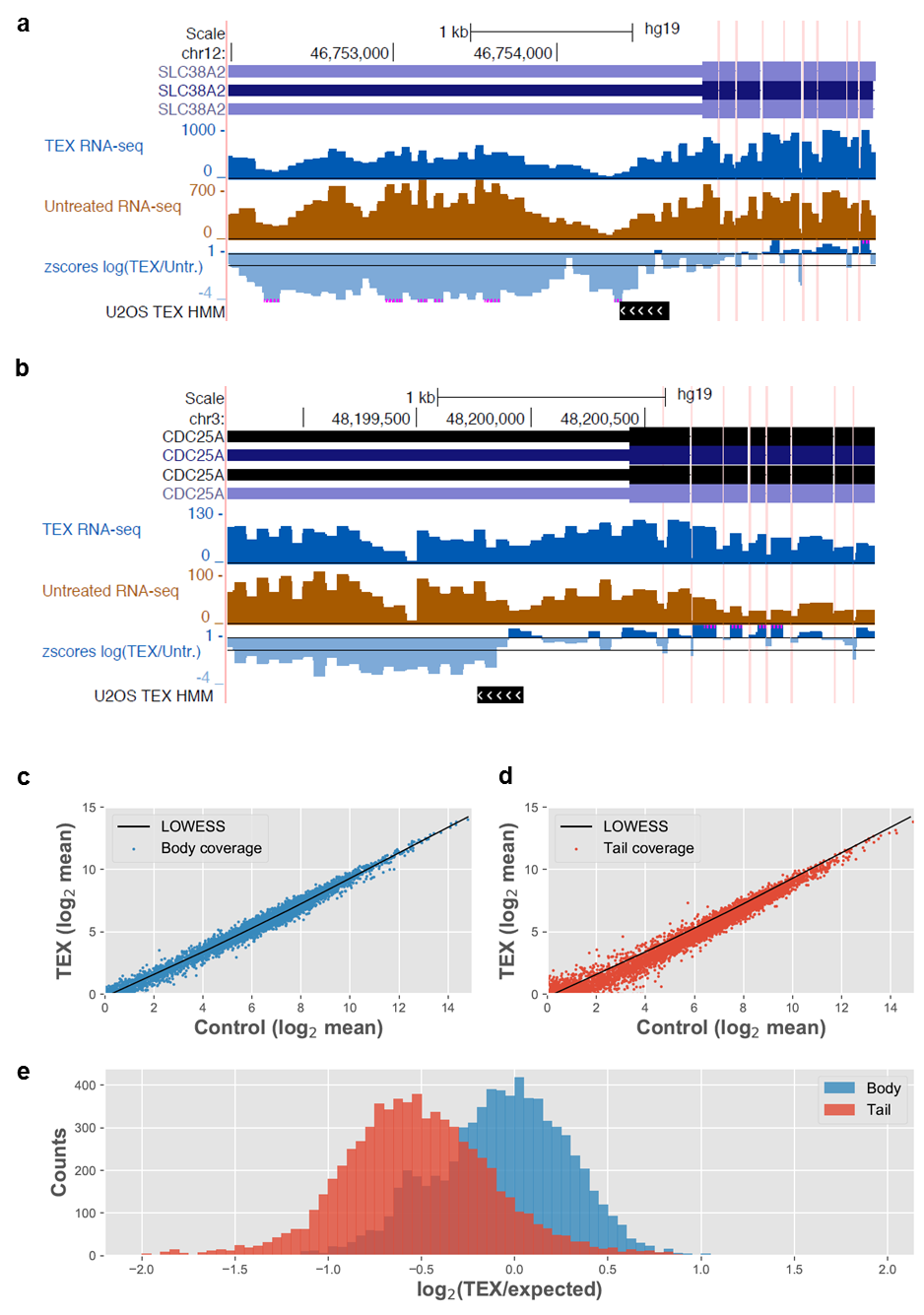 |
| --- |
| **Supplementary Fig. 7 a-b** RNA-seq read coverage following TEX treatment (blue) or untreated (brown) for SLC38A2 (**a**) and CDC25A (**b**) genes in U2OS cells. Introns are hidden (marked by vertical pink lines). Also shown (bottom of each plot) are the z scores of the log ratio between the two conditions (calculated based on body). Horizontal lines mark +/- 1 s.d.. A black rectangle marks the predicted position of cleavage point, following which the z scores tend to deviate away. **c-d** Gaussian HMM was used to evaluate TEX/RNA-seq ratio along the transcript to determine cleavage point by which difference in the TEX compared to RNA-seq in U2OS cells was observed. Then transcripts in which the breakpoints fall upstream of the coding end site were filtered. By breaking the transcript to body and tail segments, before and after the cleavage point respectively, we note the difference in the TEX/RNA-seq: (**c**) TEX compared to control in the body segment. **d** TEX compared to control in the tail segment. **e** Comparing the expected TEX according to the control to the actual TEX in body and tail segments.   \| 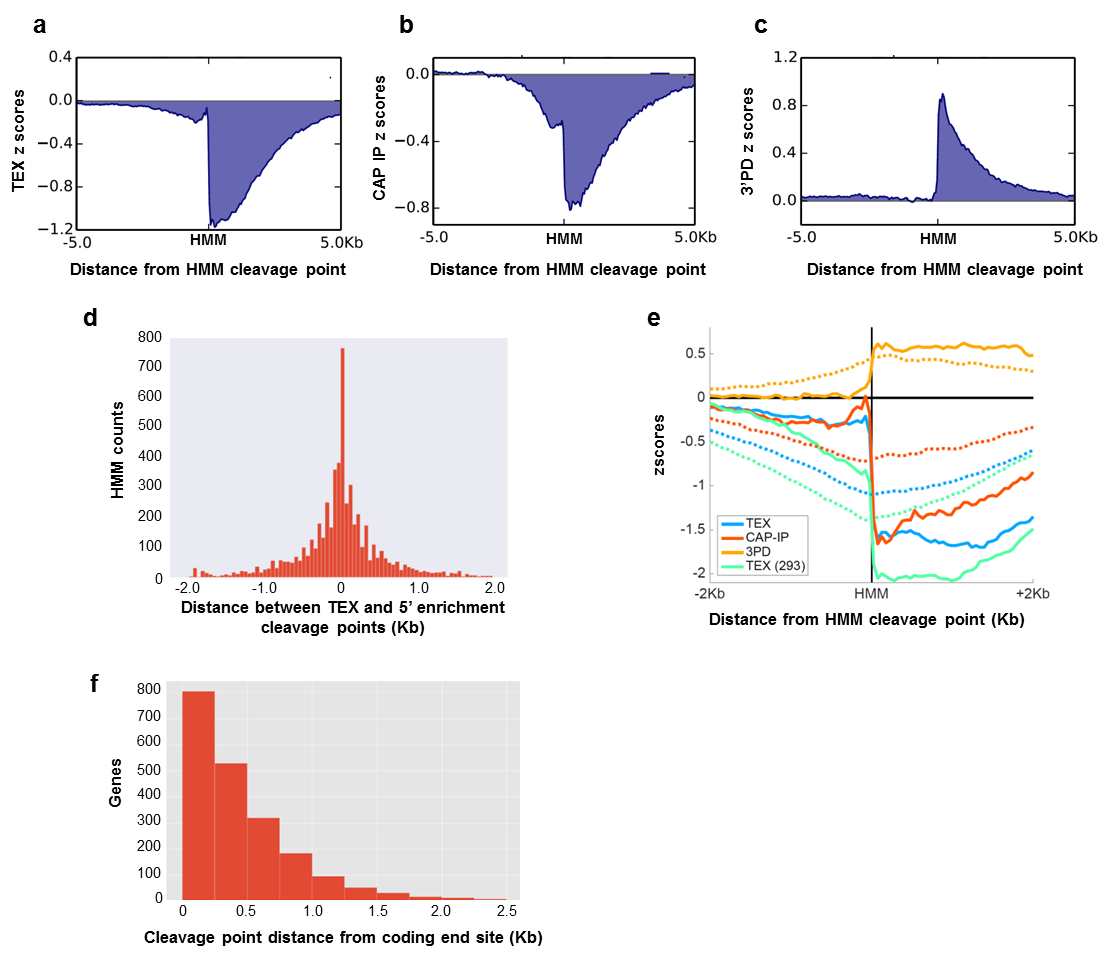 \| \| --- \| \| **Supplementary Fig. 8 a-c** Meta-gene analysis of normalized read coverage ratio (standard deviations from “body”) of cells treated with TEX (**a**), CAP IP (**b**), or 3’PD (**c**), centered around HMM predicted cleavage site in TEX treated cells. Tail units (right) show reduced coverage relative to body in TEX and CAP IP, or increased coverage in 3’PD. **d** Distance between TEX and 5’ enrichment HMM points. **e** For each of the four treatments (TEX, CAP IP, 3’PD in U2OS cells, and TEX in HEK-293 cells), we aligned all statistically significant UTRs (longer than 2Kb) by the predicted HMM cleavage point, and computed the average z scores in +/-2Kb windows. As shown, all treatments tend to show near average signal (z score ~ 0) upstream, with sharp deviations following the predicted cleavage point. These include elevated RNA-seq coverage following 3’PD, or weaker RNA-seq coverage following TEX and CAP IP. Dotted lines correspond to similar analysis using random positions in random UTRs (longer than 2Kb). **f** Distribution of distances of TEX HMM points from coding end site. \|  \| 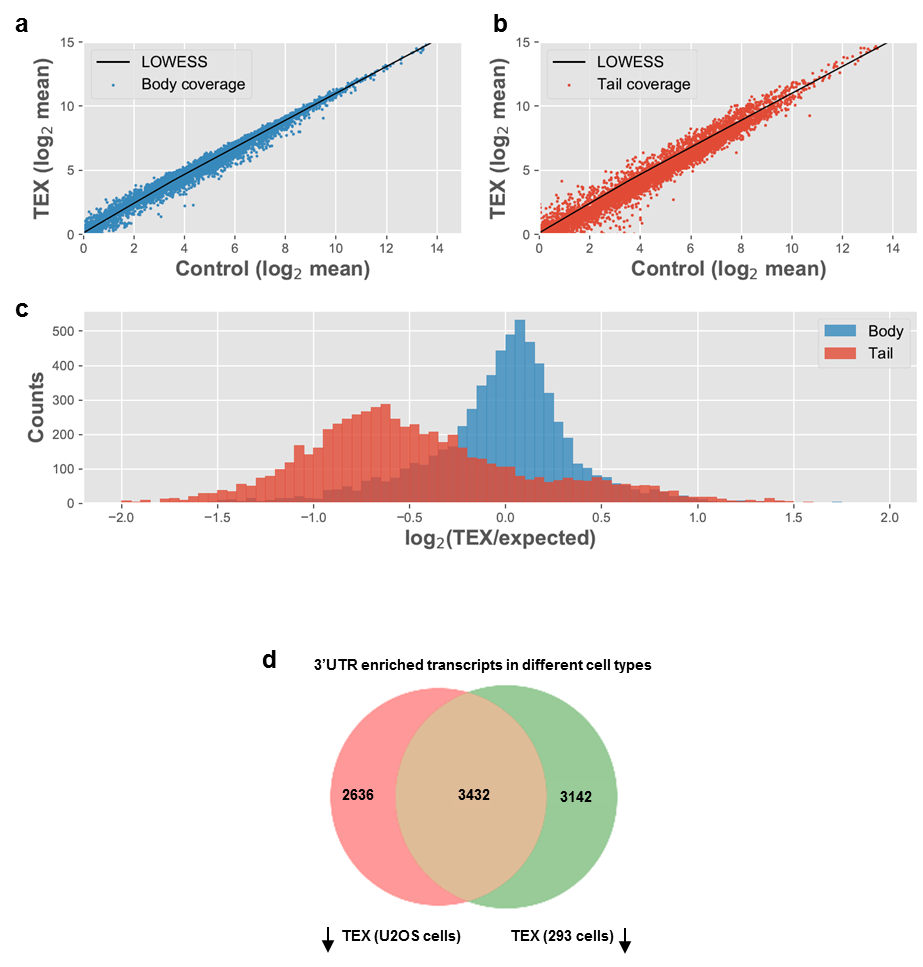 \| \| --- \| \| **Supplementary Fig. 9** **a-b** Gaussian HMM was used to evaluate TEX/RNA-seq ratio along the transcript to determine cleavage point by which difference in the TEX compared to RNA-seq in HEK293 cells was observed. Then transcripts in which the breakpoints fall upstream of the coding end site were filtered. By breaking the transcript to body and tail segments, before and after the cleavage point, respectively, we note the difference in the TEX/RNA-Seq: **a** TEX compared to control in the body segment. **b** TEX compared to control in the tail segment. **c** Comparing the expected TEX according to the control to the actual TEX in body and tail segments. **d** Venn diagram comparison of genes found to differ (p < 0.01 with FDR correction) in their body/tail coverage distribution for TEX relative to the control in U2OS and HEK293 cells. \| |

| 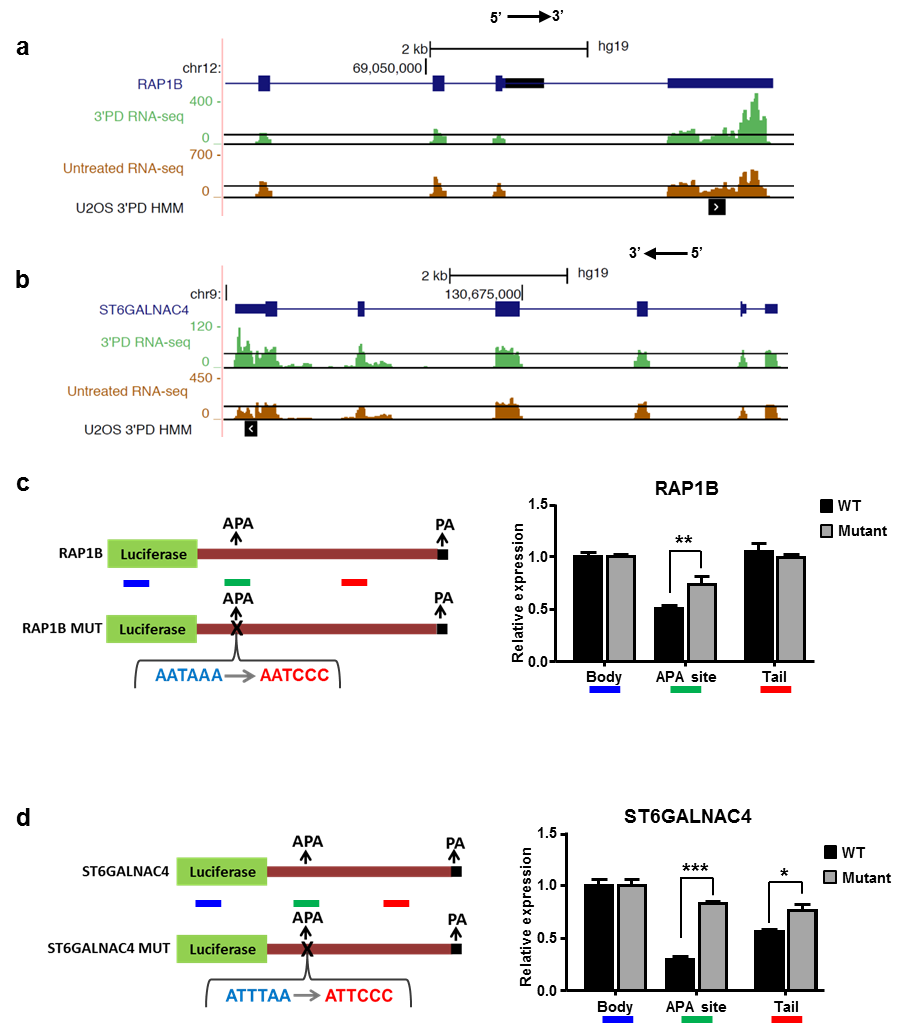 |
| --- |
| **Supplementary Fig. 10** 3’UTR of RAP1B (**a**) and ST6GALNAC4 (**b**) was cloned into pGL3 promoter luciferase reporter system. For each clone, we mutated the APA site and used 3 sets of primers for qRT-PCR analysis for amplifying body, tail and the APA site. **c-d** Relative expression of the APA indicates a significant decrease in expression in the cytoplasm RNA fraction in the WT 3’UTR (black bars) compared to the mutated clones (gray bars). Data are normalized to the first sample (Body of WT). *P < 0.05, **P < 0.01 and ***P < 0.001 (two-tailed Student’s t-test). Error bars represent s.d. of three independent experiments **(c, d)**. |

| 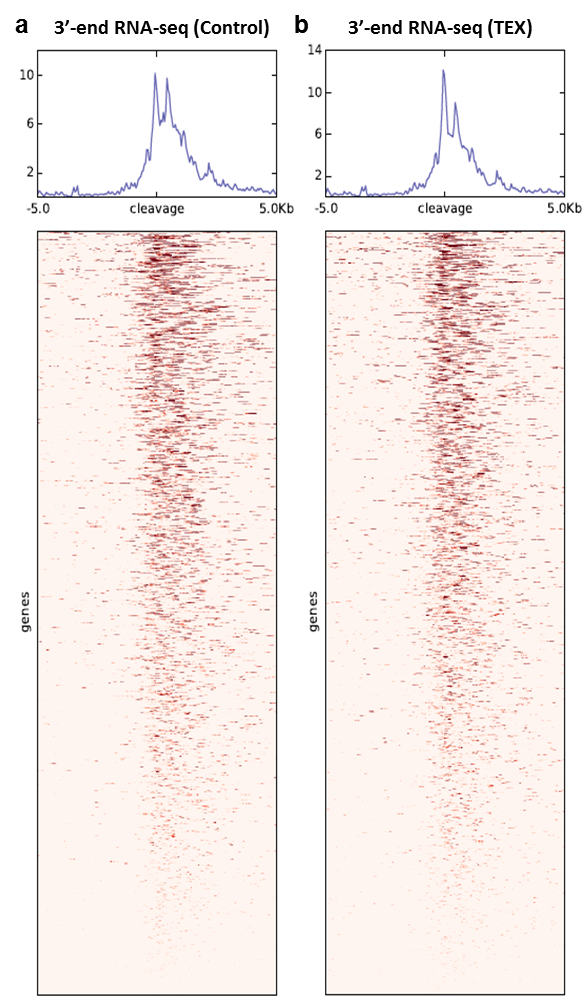 |
| --- |
| **Supplementary Fig. 11 a-b** Meta-gene analysis and heatmaps showing 3’-end RNA-seq read coverage around HMM predicted cleavage points for untreated (**a**) and TEX treated cells (**b**). Note relative decrease in strength of distal 3’-end peak following TEX. |

| 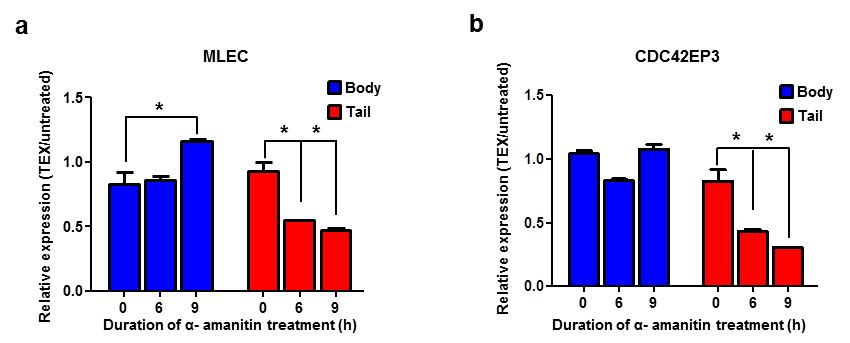 |
| --- |
| **Supplementary Fig. 12 a-b** U2OS cells were treated with α-amanitin (10µg/ml) for 6 or 9 hours or left untreated. Cytoplasmic RNA was then treated with TEX and subjected to qRT-PCR analysis using two sets of primers for the body or the tail of MALC (**a**) and CDC42EP3 (**b**). Histograms represent relative expression in the TEX treated RNA versus relative expression in TEX-untreated RNA. Expression levels were normalized to UBC. *P<0.05 (two-tailed Student’s t-test). Results are representative of three independent experiments. Error bars, s.d. |

| 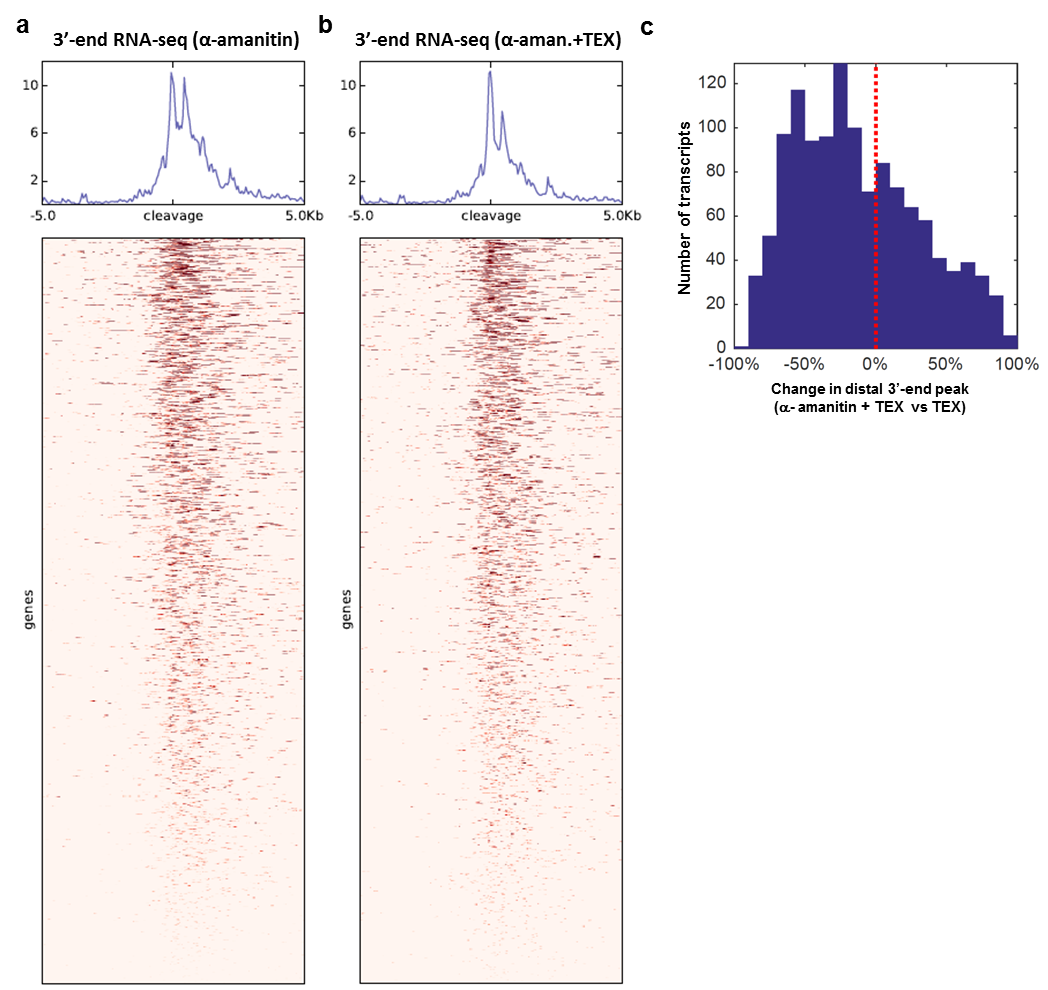 |
| --- |
| **Supplementary Fig. 13 a-b** Meta-gene analysis and heatmaps showing 3’-end RNA-seq read coverage around HMM predicted cleavage points for RNA from α-amanitin treated cells; TEX-untreated (**a**) and TEX-treated (**b**). Note relative decrease in strength of distal 3’-end peak following TEX in α-amanitin treated cells**. c** Histogram showing the relative change of distal peak heights (3’-end RNA-seq) of TEX treated RNA from cells treated by α-amanitin for 9 hr vs TEX treated RNA from cells not treated with α-amanitin (p< 4.8e-6, 1484 transcripts). |

| 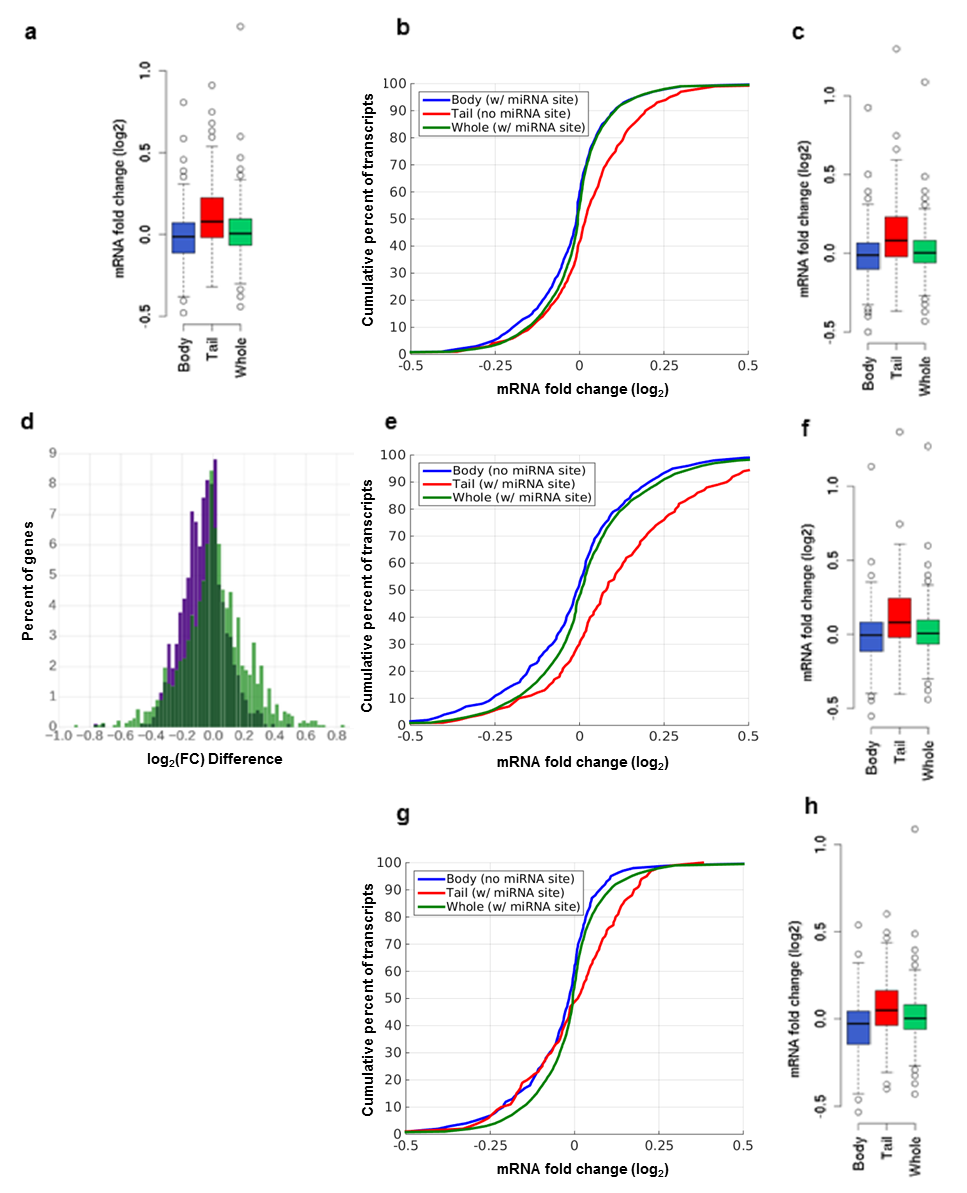 |
| --- |
| **Supplementary Fig. 14** HEK293 transfection experiments. **a** Boxplot of expression changes (log_2_ fold change) in transcripts with miR-92a 7-mer seed binding sites in the body, corresponding to figure 4c. **b** Cells were transfected with synthetic let-7a/control double-stranded RNA (dsRNA), and total RNA was extracted 40 h after transfection. Cumulative frequency distributions of expression changes in transcripts with let-7a 7-mer seed binding sites in the body are shown (log_2_ expression fold change, log_2_(FC), values are presented). Comparisons were conducted by a two-sided Kolmogorov–Smirnov test. **c** Boxplot of log_2_ fold change values of the data presented in **b**. **d** Histograms of log_2_ fold change differences between the body and corresponding tail transcript region in transcripts where a cleavage site was identified (purple), between the coding sequence (CDS) and its corresponding 3′ untranslated region (3′UTR) in transcripts where no cleavage site was found (light green), and in the overlap between them (dark green). In all transcripts, a let-7a binding site was located exclusively in either the body/CDS of the transcript, or in the tail/3′UTR. **e-h** Cumulative frequency distributions (**e** and **g**) and corresponding boxplots (**f** and **h)** of expression changes in transcripts with miR-92a (**e, f**) and let-7a (**g,h**) 7-mer seed binding sites in the tail, in which results were considered to be inconclusive. In both **e** and **g** the distribution of log_2_ fold change between the body and the tail was statistically significant (p ≤ 2.9e-11 and p ≤ 5.3e-7, respectively). In both **(e)** and **(f)**, the tail did not show a larger decrease in expression than the body, as would be expected, whereas in **(g)** and **(h)** the distribution of down-regulation between the body and tail was similar. Potential inconsistencies could be attributed to several sources: Firstly, mammalian genes have more than one APA site, such that it is difficult to predict the intactness of the RNA fragment. Our analysis was based on the first cleavage point found computationally following TEX treatment. However, if multiple cleavages occur, it could be that the actual tail begins downstream of the putative cleavage point. Hence the predicted miRNA binding site might not be within the actual tail transcript, such that down-regulation would not be expected, which could explain the discrepancy observed. Moreover, our analysis cannot discern if reads upstream or downstream of the cleavage site are body/tail transcripts or belong to transcripts that were not cleaved and remain whole, and always assumes the former. When the miRNA binding sites were found in the tail region down-regulation of the un-cleaved, whole transcript is expected, as the miRNA site occurs in its 3’UTR, but since our analysis considers these reads to be part of the body, we observe the expression of the body to be decreasing relative to the tail as a result of the miRNA transfection, despite the body having no miRNA binding site (blue versus red plot). |

**Supplementary Note 1:**

**Sequence of cloned 3’UTRs**

**ST6GALNAC4 UTR:**

CTTCCGTCGTCCTGCCAGTCGCCATGCCGTTGCGAGGCCTCCGGGATGTC

CCATCCCAAGCCATCACACTCCACAAAAAC**ATTTAA**TTTATGGATCCTGC

CTCCTGCCACGTGCTGGGTGGACCTAAGGTTCCTTCCCGCCCCATTCTGG

CGACACTTGGAGCCATCTCAGGCCTCATGACTTGAAGGGGAGTGGAGGGG

GGAGCCGTGTCTCCCCCCTCCACTCCCTGAGTAATTCACGGCATTTGGGG

GCTCACCCCACCTCCAGGTCTGTCAAGTGGCCTTTGTCCCTGGGGCTGAT

GGCCCCCAACTCACCAGCATCATGACCTTGTGCCAGTCCTGGTCCTCCCT

CCCCAGCCGCTCCTACCACCTTTTGGTGCCACACTTCTCAGGCTGGCCGC

CCTGGTTGGGGCAGCCGAGAGCCTGGGGTTCATTGGTGAAGGGGCCTTGG

AGTTGTGACTGCCGGGGCCGTATCAGGAACGTACGGGTAAACGTGTGTTT

TCTGGA

**ST6GALNAC4 mutant UTR:**

CTTCCGTCGTCCTGCCAGTCGCCATGCCGTTGCGAGGCCTCCGGGATGTC

CCATCCCAAGCCATCACACTCCACAAAAAC**ATTCCC**TTTATGGATCCTGC

CTCCTGCCACGTGCTGGGTGGACCTAAGGTTCCTTCCCGCCCCATTCTGG

CGACACTTGGAGCCATCTCAGGCCTCATGACTTGAAGGGGAGTGGAGGGG

GGAGCCGTGTCTCCCCCCTCCACTCCCTGAGTAATTCACGGCATTTGGGG

GCTCACCCCACCTCCAGGTCTGTCAAGTGGCCTTTGTCCCTGGGGCTGAT

GGCCCCCAACTCACCAGCATCATGACCTTGTGCCAGTCCTGGTCCTCCCT

CCCCAGCCGCTCCTACCACCTTTTGGTGCCACACTTCTCAGGCTGGCCGC

CCTGGTTGGGGCAGCCGAGAGCCTGGGGTTCATTGGTGAAGGGGCCTTGG

AGTTGTGACTGCCGGGGCCGTATCAGGAACGTACGGGTAAACGTGTGTTT

TCTGGA

**RAP1B UTR:**

TATACTAAATGCATTGTAGCTCTGAGCCAGGTCTGAAGAACTGTTGCCCA

ATTCAACAGTGCCAGCATTCCAACTTTGTTAAACCTACCAACATCTTAAA

TGGACTTTCCTGTGGTGGTACCCTTTAAGAGGCGGATGAAAGCTACTATA

TCAGTTTGCACATTCTAATCACTTTCCAGTATCACAAGAGAGATTTTTAC

TTATATAATAGTCCTAGAGTTTGCAGCTGGTAAAACCAGAGGCTACATCC

AGTATTACTGCTAAGAGACATTCTTCATCCACCAATGTTGTACATGTATG

AAAATGGTGTACTGTATACTTTAACATGCCCCATACTTTGTATTGGAGAG

TACAATAATGTAAATCCTAAAAGCACCACTATTTTAGCAT**AATAAA**AGAA

AGTCCAAAGAGCTCCTATATAGACTACTCCAGATAACTTCGCTTCTTTGA

TACTTGTAGCTTATTGTAATTTTTTTTAAGAAATTCAAGGTCATTATTAT

TGTACAAAATAAGCGCTTTGATTAACACAGCTATATAGTTTTTTTAATTT

TTAAAAAACCTGTGGAGACGGTGATCTTGTCTTTAAAACATGATAGTCCT

TTCAGTATAATGTCTTAGATTAAAGACGTTGCCTTTAATATCTGTTGGGA

AGGAAATGTCCAGACTTTTCAAATCTCTTATTATATGTTTCCTTTTTTTG

TTTACATAGGGAACAATGTTTATAGTCGTGTGTACAGTGGGGGTCTACAA

CAAGAAGTGTATATTTTCAAACAATTTTTTAATGATTTAACAATTTTTGT

AAATCATTTTCAGGCTTCTGCAGCTGTAGATTCTCACTGTGAATCCCTTG

CTTGCTCATGCATAAGTGTATTTGCAATACCAAATATACAGGTTTAGTAT

TTTTGCCTGTTAGTGATTGTTTCACATGTGTAACGTTTTGGTTGAGATGT

TAAATGGTGGACGAGTACTGTGGATGTGAATGTGGGAAGTAATTTTAATC

ATATGTAATTGGTCACAAGGCCTAATTTGCAGTAACTATTGCTGTTTTAT

TTAACAATGCCTTGTTGCTTTGTATGCATTAATGTTTGGATGTAAAGATT

GTGTGTCTATCCAACAGGGAGCCACAGTATTTAAATTGACCAACCTAATG

TTACAACTACTTTGAGGTGGCCAAATGTAAACTAAAAGCCTTAATTAAAG

TGGTGCAATTTTGTATAACTTAGCATCAGTAGTTCAATAAATTTGGATTG

CCATGCAAGGGCTTGCATTATAA

**RAP1B mutant UTR:**

TATACTAAATGCATTGTAGCTCTGAGCCAGGTCTGAAGAACTGTTGCCCA

ATTCAACAGTGCCAGCATTCCAACTTTGTTAAACCTACCAACATCTTAAA

TGGACTTTCCTGTGGTGGTACCCTTTAAGAGGCGGATGAAAGCTACTATA

TCAGTTTGCACATTCTAATCACTTTCCAGTATCACAAGAGAGATTTTTAC

TTATATAATAGTCCTAGAGTTTGCAGCTGGTAAAACCAGAGGCTACATCC

AGTATTACTGCTAAGAGACATTCTTCATCCACCAATGTTGTACATGTATG

AAAATGGTGTACTGTATACTTTAACATGCCCCATACTTTGTATTGGAGAG

TACAATAATGTAAATCCTAAAAGCACCACTATTTTAGCAT**AATCCC**AGAA

AGTCCAAAGAGCTCCTATATAGACTACTCCAGATAACTTCGCTTCTTTGA

TACTTGTAGCTTATTGTAATTTTTTTTAAGAAATTCAAGGTCATTATTAT

TGTACAAAATAAGCGCTTTGATTAACACAGCTATATAGTTTTTTTAATTT

TTAAAAAACCTGTGGAGACGGTGATCTTGTCTTTAAAACATGATAGTCCT

TTCAGTATAATGTCTTAGATTAAAGACGTTGCCTTTAATATCTGTTGGGA

AGGAAATGTCCAGACTTTTCAAATCTCTTATTATATGTTTCCTTTTTTTG

TTTACATAGGGAACAATGTTTATAGTCGTGTGTACAGTGGGGGTCTACAA

CAAGAAGTGTATATTTTCAAACAATTTTTTAATGATTTAACAATTTTTGT

AAATCATTTTCAGGCTTCTGCAGCTGTAGATTCTCACTGTGAATCCCTTG

CTTGCTCATGCATAAGTGTATTTGCAATACCAAATATACAGGTTTAGTAT

TTTTGCCTGTTAGTGATTGTTTCACATGTGTAACGTTTTGGTTGAGATGT

TAAATGGTGGACGAGTACTGTGGATGTGAATGTGGGAAGTAATTTTAATC

ATATGTAATTGGTCACAAGGCCTAATTTGCAGTAACTATTGCTGTTTTAT

TTAACAATGCCTTGTTGCTTTGTATGCATTAATGTTTGGATGTAAAGATT

GTGTGTCTATCCAACAGGGAGCCACAGTATTTAAATTGACCAACCTAATG

TTACAACTACTTTGAGGTGGCCAAATGTAAACTAAAAGCCTTAATTAAAG

TGGTGCAATTTTGTATAACTTAGCATCAGTAGTTCAATAAATTTGGATTG

CCATGCAAGGGCTTGCATTATAA

**Supplementary Note 2:**

**5’RACE product sequencing data:**

**RED** – APA site

**BLUE** – 5’RACE sequence

**Mknk2 3’UTR:**

5'……GGGTGGGGGCCTGGGACACCCACTGCCCAG**CATCCTCCACCCTCCT**

**CCCTAGTCTCATAGGATCGTCACAGTGGAGGTGACATGCCTTCTCCAGTC**

**CTGCCCCACCTGCCTCTGTGGACACATTTCCAAAGAACCCCTGGGGGTGG**

**GACCTCCTCCATCAGTATGACTCAGCTGTTGGCCACCTGAGGACTCGGCC**

**CCCCTGCAGGTTCCTGAAGCAACCTGACTGGGCAGTGAGCAGCATTGACC**

**CCCACTCACCCCCAAAACAGGG**CTGTGATTTCCTTAGTCCTTCCAA……3'

**Ssr1 3’UTR:**

5'……TTTTTTTTTTTAAAAAACCATTAAA**CTGTGTGGGTGTGGATGTCTG**

**TGAGCAGTTGTCTTAC**C**AGAATCAATGTTAAACTACCTGAAACACACCTT**

**TAGCATACTCAGCAAAACGCTCTCGCCACTTCCCTTATGCTAGTTTCCTG**

**TTTTGACCCCAAGTTCAATTAATATTTTTTTCCCAGTGTGGTTTTAGGTG**

**AATTGTTTTGAGGGAATAATTTGTTTATTTCTTGGAATCGATCAAATCCA**

**AACTGAGGGCACAAGCCGGCCTTCTGCTTGCTGTCTGTCCCAGCTTGTAT**

**CTAACTGCACAGCAGGGGTG**GCTGCTGTGAGCATCAGAGGCATGCT……3'

**Bcl2 3’UTR:**

5'……GCTTTCAAAAA**GAATCCATGCATCTC**AGC**ATTGTTTAAATTGTATT**

**TAGTTATGACCTCTAGAATGTTTGTTAGTCACACAAACAGAAAAAGATAC**

**TTTTTCCATTTGAGGTATTTGTCTCTTGATTCTTCAAAAGCATTTCTGAA**

**AAGGTGG**GAACAGCTTTAGAGAGATGCGAGGAACCGATGGAGCTGG……3'

**Rab2a 3’UTR:**

5'……AAGCATTGTTAGGAATTGCTTGGACACTGACTTTTCAACTTTTTGA

CATCG**T**TAACGA**GCATGTTCATCTTTTCTTGTCACTAGTCCAAGAAAACT**

**ACGCTTCCTGTGTATTACTAAAGGCTGTGTGTGTGTTAACCTGTTTTAAT**

**GCCAAAAGCTATTTGTCCACAAGTTCTTGAGACCGCTTCACAAAAGGATT**

**TGTTTGCCTTAATGAGTGCTGTTGGGTAAAACACAGTGTAATGAGTGGGA**

**CGGGCAGAAACAGAAACTCCAACACTACACAACTCCCAAGAGCAAGGCCC**

**ACTCGGAGAAGGCACGCCATGCCACTTACTCTTCCCTTAGACACAATAAT**

**GGTCCCTTTTCTTCACACAGTTTCTAAGTA**TATTTTCTTGCAGGGC……3'

**Gid4 3’UTR:**

5'……ATCTCTTTGTCTTCAGGGTCTCACTTTGTAACTCTAGCTGGCCTGG

AGCTCACTTTGTAGATCAGGCTGACCTTGAACTCACTGAGATCCACCTGA

CTACTCAGAGCGAGTACTAGGACTAAAGGTGTGAGCCACCACACCCTGCT

TTTTTTTTTTTTTTTTTTCTTAGCTA**G**GGAAGTGTTCC**AGCTCTACTTAG**

**AAAGATGAGGGAAGGATGCCTTTAGCAGACAGACTTCAGACCTACCTGAA**

**GACTTGCTGCTTAGAAGTCTGCCCCTGAACACTGGGCAGGAATAAACATG**

**TTCCACTTCTTAGTAACTCCTAGCTGGTGGACAGTGACTTCTTTTCCTTT**

**TCTGTCCCTGGAGGACCTCTGGGACTTGTCTCCATTGGTAGCTCTTTAAC**

**CAGCTGCTCAGTGGCTTGCATGCCTCTGGTCCTGTGTGCAATATCTTTGC**

**CTGGCACTTGTATCTAACATTGAAAAATAGGTGCAGACTGCAAATGTCCT**

**ACTGTTCTCTACTCCAACGAGCCAACCCCCAGGCAGGGACTAGAGGTCTG**

**GCTGCTCTTCTCTTACCCTCCACTCAGAAGGCTCCACTGCTGTGGCCAGC**

**TGTGGCCCTCTCGTTCCCTGCTTTACCAGTCTACCCACCTATCTCCTTTA**

**AGATGGCCTAGCATGTCAGCTGCAGTCCAAAAGTAAGTAGACAAAGCCTC**

**ATGTTAAGTAGACAGTCAGGGTGGACTGGCCCTGTTAGCAGTGTTGGAGT**

**CAGATTCCAGTGGAACAAAGGGGTT**TCTCAATGTCTTTTGCTTTCA……3'

**Ifngr2 3’UTR:**

5'……GTAATTTAAGTCTTTTTTTTTTTTTTTTAATCTAGAGATAAAAGAT

TGTAT**GTTAAACTTTT**TTTTTTTTTAAGTCTATCT**GCCGGGTGGTGGTGG**

**TGCACACCTTTACTCCTGGCACTTGGGAGGCAGAGGCAGGAGGATCTCTG**

**TGAGTTCGAGGCCAGCCTGGTCTAGAGTCCTGGTCTAGGACAGCCAAGGC**

**TACACAGAGAAACCCTGTCTGGTAAACAAAACAGGAAAAAGAAGTGCACG**

**TGTTTCTTCTTTTCTTGTCCCTTACCACGTCGCCTTCTTTGCCTGTTTTC**

**CGTAAAGTATTGACTTGTGAAAAGTCTTAGCCAGGCATGGTGACACATGC**

CTTTATTCCCAGCACTTGAAAGCAGAGGCCAGTAGATTTCTGTGAG……3'

**Supplementary Note 3:**

**miRNAs sequence:**

**Negative control:**

rCrGrUrUrArArUrCrGrCrGrUrArUrArArUrArCrGrCrGrUAT

rArUrArCrGrCrGrUrArUrUrArUrArCrGrCrGrArUrUrArArCrGrArC

**has-miR-92a-3p:**

Sense: /5Phos/rUrArUrUrGrCrArCrUrUrGrUrCrCrCrGrGrCrCmUmGrU

Anti-sense: mAmGrGmCrCmGrGmGrAmCrAmArGmUrGmCrAmArUA

**has-let-7e-5p:**

Sense: /5Phos/rUrGrArGrGrUrArGrGrArGrGrUrUrGrUrArUrAmGmUrU

Anti-sense: mCmUrAmUrAmCrAmArCmCrUmCrCmUrAmCrCmUrCA

**Supplementary Tables:**

**Supplementary Table 1:** Primers list for qRT-PCR from mouse cells

|  | Forward | Reverse | Amplicon |
| --- | --- | --- | --- |
| Bcl2 5’ | AGGAGCAGGTGCCTACAAGA | GCATTTTCCCACCACTGTCT | 166bp |
| Bcl2 cleavage | TTTGGTTCTATTTGAAAATCTGACA | TGAACTTGGGGAAAAAGTGG | 197bp |
| Bcl2 3’ | TGGGTGTGGCCTTTATTCAT | TGGGTACTGCCACCCATTAT | 177bp |
| Rab2A 5’ | TACCAACGCATCTCATGGAA | GCTGAGCATGAGGAAAGAGG | 129bp |
| Rab2A cleavage | TGAAATGGCTTTATGTCACAGG | TTGACCTAGAAATCAAATGCAAA | 136bp |
| Rab2A 3’ | GAGACCGCTTCACAAAAGGA | GGGAAGAGTAAGTGGCATGG | 158bp |
| Ssr1 5’ | ATGTTGTGTGTGCCGCAGT | ATCGTATGGCCACAGACTCC | 147bp |
| Ssr1 cleavage | TGCACTGCATTGCTTTTCTAA | TGCTAAAGGTGTGTTTCAGGT | 149bp |
| Ssr1 3’ | AAGAGGCTTCCTTCCCTCTG | GTGTGTGGAAGGGATTGGAC | 135bp |
| Ubc | GCCCAGTGTTACCACCAAGA | CCCATCACACCCAAGAACA | 104bp |

**Supplementary Table 2:** Primers list for qRT-PCR from human cells

|  | Forward | Reverse | Amplicon |
| --- | --- | --- | --- |
| MLEC 5’ | CAATAAGAACCGGGTGCAGT | AGGCAGAAGAGGGTTGGAAT | 120bp |
| MLEC 3’ | GGCAGCCTCTTCAACCATTA | GCCTCCACAGAGAGAGGATG | 169bp |
| SLC38A2 5’ | TACTGGTGATGACCGGAAGC | CAACACTGGCATCAGATGGA | 120bp |
| SLC38A2 3’ | CATGAAATCTTGGTACAAATTCAGA | CACCCAGGTTCTCAAAGGTC | 144bp |
| CDC42EP3 5’ | TTCCAAACAGGAGTCCTTCG | GTGTCTCCCTGGTGGACTGT | 123bp |
| CDC42EP3 5’ | TTCATCCAGCAAGCTTTTCA | CCACATGATGCTTTTTGCAG | 136bp |
| CDC25A 5’ | ACAACCGATGCAAGCTGTTT | CTCATGGGCCTTCTCTGGAT | 173bp |
| CDC25A 3’ | TCAACTTCTTGGGGTGGGAA | GAATCTGTCTCAATGCGCGT | 145bp |
| Luciferase | TTGTGCCAGAGTCCTTCGAT | TAGGATCTCTGGCATGCGAG | 135bp |
| RAP1B 3’ | ACTGTGAATCCCTTGCTTGC | TCCACAGTACTCGTCCACCA | 159bp |
| ST6GALNAC4 3’ | CCACCTCCAGGTCTGTCAAG | GAAGTGTGGCACCAAAAGGT | 130bp |
| ST6GALNAC4 APA site | TTTATGGATCCTGCCTCCTG | CCCAAATGCCGTGAATTACT | 163bp |
| RAP1B APA site | CCTAAAAGCACCACTATTTTAGCA | TGTTAATCAAAGCGCTTATTTTGT | 162bp |
| UBC | ATTTGGGTCGCAGTTCTTG | TGCCTTGACATTCTCGATGGT | 133bp |

**Supplementary Table 3:** Primers list for 5’ RACE experiments

|  | Reverse |
| --- | --- |
| Mknk2 | GGTCGGGCTTGGAAGGACTAAGGAA |
| Gid4 | CCCTGCTTCTGAAAGCAAAAGACATTG |
| Ifngr2 | GCCTCTGCTTTCAAGTGCTGGGAAT |
| Ssr1 | CCCAGCATGCCTCTGATGCTCAC |
| Rab2a | TCAAGGTTGACAAACTGCCCTGCAA |
| Bcl2 | TCCATCGGTTCCTCGCATCTCTCTA |

**Supplementary Table 4:** Primers list for riboprobes

|  | Forward | Reverse | Amplicon |
| --- | --- | --- | --- |
| Actb CDS | GACGAGGCCCAGAGCAAGAGAGGTA | T7(TAATACGACTCACTATAGGG)GCTACGTACATGGCTGGGGTGTTGA | 233bp |
| Actb tail (3’UTR) | GGGGAAGGTGACAGCATTGCTTCTG | T7(TAATACGACTCACTATAGGG)TCCCAGGGAGACCAAAGCCTTCATA | 170bp |

**Supplementary Table 5:** Primers list cloning of 3’UTRs into pGL3 Luciferase Reporter Vector

|  | Forward | Reverse |
| --- | --- | --- |
| ST6GALNAC4 UTR | GAATCTAGACTTCCGTCGTCCTGCCAGTC | GAATCTAGATCCAGAAAACACACGTTTACCCGT |
| ST6GALNAC4 MUT UTR | CCCTTTATGGATCCTGCCTCCT | GGGAATGTTTTTGTGGAGTGTGATG |
| ST6GALNAC4 MUT OL | TCACACTCCACAAAAACATTGGGTTTATGGATCCTGCCTCCT | AGGAGGCAGGATCCATAAAGGGAATGTTTTTGTGGAGTGTGA |
| RAP1B UTR | CCCAGAAAGTCCAAAGAGCTCCTATATAGACTACCC | GAATCTAGATTATAATGCAAGCCCTTGCATGGC |
| RAP1B MUT UTR | AGAAAGTCCAAAGAGCTCCTATATAGACTACCC | GGGATTATGCTAAAATAGTGGTGCTTTTAGGATTTAC |
| RAP1B MUT OL |  | ATATAGGAGCTCTTTGGACTTTCTGGGATTATGCTAAAATAGTGGTGCTTTTAGG |
