# Supplementary material for: Post-transcriptional 3´-UTR cleavage of mRNA transcripts generates thousands of stable uncapped autonomous RNA fragments
Source: Nat Commun. 2017 Dec 11;8:2029. doi: 10.1038/s41467-017-02099-7 (PMC5725528; doi:10.1038/s41467-017-02099-7)
Supplement: Supplementary file 2 — Descriptions of Additional Supplementary Files [file 41467_2017_2099_MOESM2_ESM.pdf]

Descriptions of Additional Supplementary Files

File Name: Supplementary Data 1
Descriptions: HMM predictions for TEX, U2OS cells

File Name: Supplementary Data 2
Descriptions: HMM predictions for CAP IP, U2OS cells

File Name: Supplementary Data 3
Descriptions: HMM predictions for 3’ pulldown, U2OS cells

File Name: Supplementary Data 4
Descriptions: HMM predictions for TEX, HEK-293 cells

File Name: Supplementary Data 5
Descriptions: Analysis of 3’-end RNA-seq, U2OS

File Name: Supplementary Data 6
Descriptions: Analysis of miR-92a, HEK-293 cells

File Name: Supplementary Data 7
Descriptions: Analysis of let-7a, HEK-293 cells
